# VGF nerve growth factor inducible is involved in retinal ganglion cells death induced by optic nerve crush

**DOI:** 10.1038/s41598-018-34585-3

**Published:** 2018-11-06

**Authors:** Hiroto Takeuchi, Satoshi Inagaki, Wataru Morozumi, Yukimichi Nakano, Yuki Inoue, Yoshiki Kuse, Takahiro Mizoguchi, Shinsuke Nakamura, Michinori Funato, Hideo Kaneko, Hideaki Hara, Masamitsu Shimazawa

**Affiliations:** 1 Department of Biofunctional Evaluation, Molecular Pharmacology, Gifu Pharmaceutical University, Gifu, Japan; 2 Department of Clinical Research, National Hospital Organization, Nagara Medical Center, Gifu, Japan

## Abstract

VGF nerve growth factor inducible (VGF) is a polypeptide that is induced by neurotrophic factors and is involved in neurite growth and neuroprotection. The mRNA of the *Vgf* gene has been detected in the adult rat retina, however the roles played by VGF in the retina are still undetermined. Thus, the purpose of this study was to determine the effects of VGF on the retinal ganglion cells (RGCs) of mice in the optic nerve crush (ONC) model, rat-derived primary cultured RGCs and human induced pluripotent stem cells (iPSCs)-derived RGCs. The mRNA and protein of *Vgf* were upregulated after the ONC. Immunostaining showed that the VGF was located in glial cells including Müller glia and astrocytes but not in the retinal neurons and their axons. AQEE-30, a VGF peptide, suppressed the loss of RGCs induced by the ONC, and it increased survival rat-derived RGCs and promoted the outgrowth of neurites of rat and human iPSCs derived RGCs *in vitro*. These findings indicate that VGF plays important roles in neuronal degeneration and has protective effects against the ONC on RGCs. Thus, VGF should be considered as a treatment of RGCs degeneration.

## Introduction

Retinal ganglion cells (RGCs) are the retinal neurons transmit the visual information to the brain through the optic nerve^1^. Apoptotic degeneration of RGCs is the cause of irreversible blindness^2^. However, the mechanism of RGCs death is not unknown.

Axonal degeneration is seen in neurodegenerative disease such as Alzheimer’s, Huntington’s disease, Parkinson’s disease and amyotrophic lateral sclerosis (ALS)^3–10^. Impairment of axons often cause neural death and the onset of clinical symptom. As therapeutic strategy of these nerurological disorders, neuroprotective approaches are studied^11^. Degeneration of the optic nerve, which is made up of axons of RGCs, is the pathogenesis of optic neuritis, glaucoma, Leber’s optic atrophy, and trauma^12^. In the optic nerve crush (ONC) model, the axonal degeneration resulting in death of RGCs occurs via directly damage to optic nerve^13–16^. Neuroprotection on RGCs and their axons is aimed at inhibition of blindness and addressed for prospective strategy^17–19^.

We have reported that SUN N8075, a free radical scavenger, reduced the loss of RGCs induced by exposure of the retina to *N*-methyl-D-aspartate^20^. We also found that SUN N8075 was able to induce the expression of VGF nerve growth factor inducible (VGF), and it was found to be neuroprotective against the endoplasmic reticulum (ER) stress-induced cell death^21^. VGF is a polypeptide induced by nerve growth factor (NGF) in rat pheochromocytoma (PC12) cells^22^. VGF is also induced by BDNF in hippocampal neuronal cells^23^. In neuronal and neuroendocrine cells, VGF peptides including NERP-1, NERP-2, TLQP-21, LQEQ-19, and AQEE-30 are processed by the prohormone convertases^24–26^. These peptides have been shown to regulate neuronal activity such as synaptic plasticity, neurogenesis, and neuritis growth.

AQEE-30 suppressed the death of STHdhQ111 cells in a Huntington’s disease model^27^. Earlier studies showed that the mRNA of the *Vgf* gene could be detected by *in situ* hybridization in adult rat retinas^28^. It has also been reported that a VGF peptide has neuroprotective effects in primary retinal cells^29^. However, the role of VGF in RGCs death has not been determined.

Thus, the purpose of this study was to determine the role played by VGF on the degeneration of the RGCs and optic nerve of mice after the optic nerve crush (ONC). In addition, we evaluated the effect of AQEE-30, a VGF peptide, on the retinal injury induced by the ONC and also on RGCs in culture.

## Results

### Level of expression of mRNA of *Vgf* gene and VGF protein after ONC

**Figure 1:**
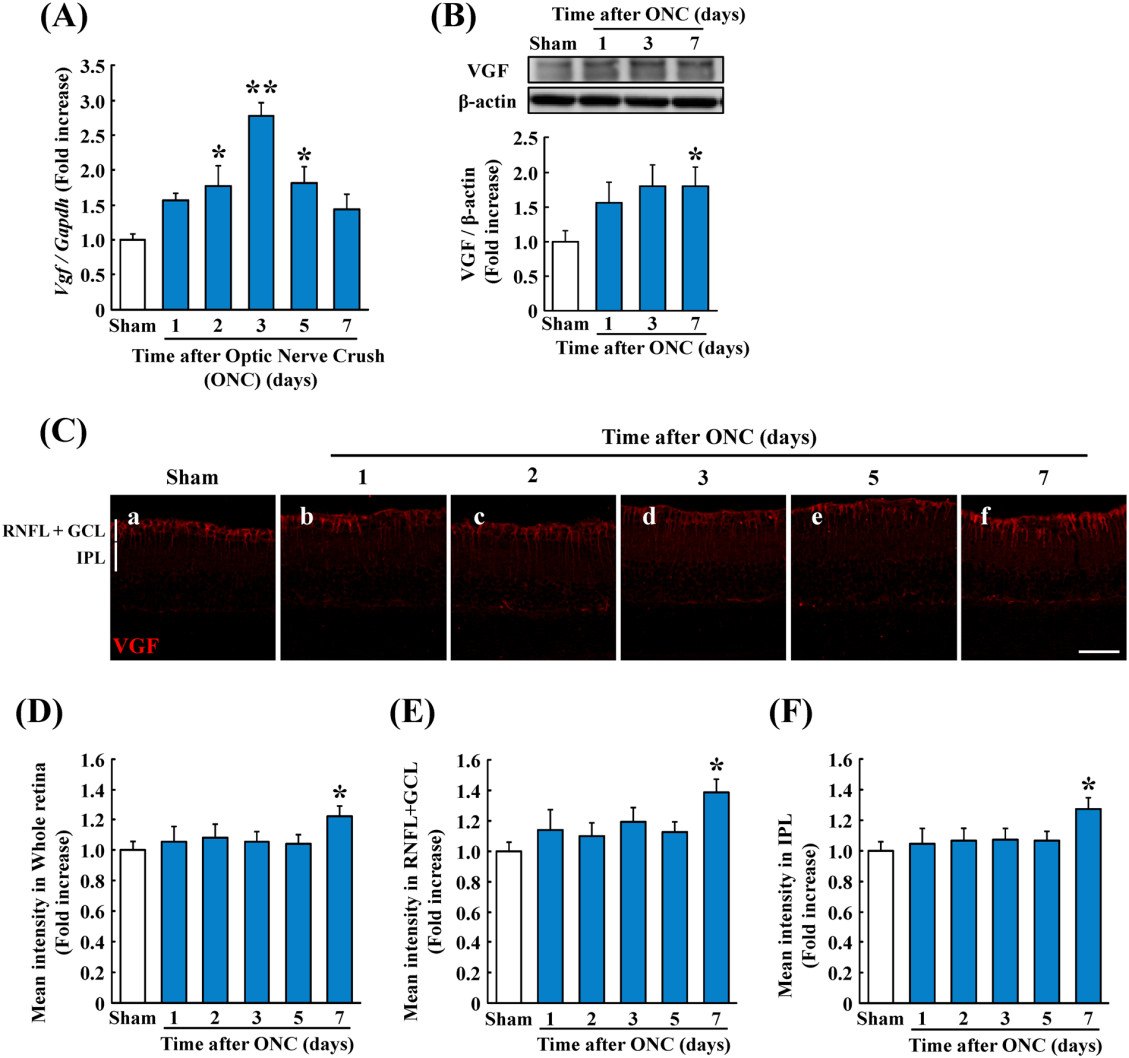
Expression of the mRNA of the *Vgf* gene and VGF protein in the optic nerve crush (ONC) model. (**A**,**B**) The quantitative data of the level of expression of *Vgf* mRNA and VGF protein after the ONC are shown. qRT-PCR was used to determine the level of expression of *Vgf* mRNA. The mRNA of *Vgf* is increased significantly at 2, 3 and 5 days after the ONC. Western blotting was used to determine the level of expression of VGF protein. The protein of VGF is increased significantly at 7 days after the ONC. Data are the means ± standard error of the means (SEMs). (A: n = 5–7, B: n = 7–12). *P < 0.05, **P < 0.01 versus sham group (A: Dunnett’s test, B: Student’s *t*-tests). (**C**) Representative images of immunostained retinal sections to determine the site of VGF protein expression after the ONC. Scale bar = 50 μm. The measurements of the intensity of the (**D**) whole retina, (**E**) retinal nerve fiber layer plus ganglion cell layer (RNFL + GCL), and (**F**) inner plexiform layer (IPL) were performed. The VGF protein increased significantly in all areas 7 days after the ONC. Data are the means ± SEMs. (n = 4 or 5). *P < 0.05 versus sham group (Student’s *t*-tests). In this figure, the cropped blots are used. The full-length blots are showed in Supplementary Fig. 1.

Initially, we determine the level of expression of the mRNA of the *Vgf* gene and the protein after the ONC. The level of the mRNA of *Vgf* was increased in the retina at 2, 3, and 5 days after the ONC (Fig. 1A). The VGF protein expression in retinas was also increased (Fig. 1B–D). Immunostaining showed that the expression was observed in the RNFL + GCL, IPL, INL, and OPL, and the degree of expression in the RNFL + GCL and IPL was higher than that in the INL and OPL at 7 days after the ONC (Fig. 1C,E,F).

### Retinal site of VGF expression in sham group and in ONC group after 7 days

**Figure 2:**
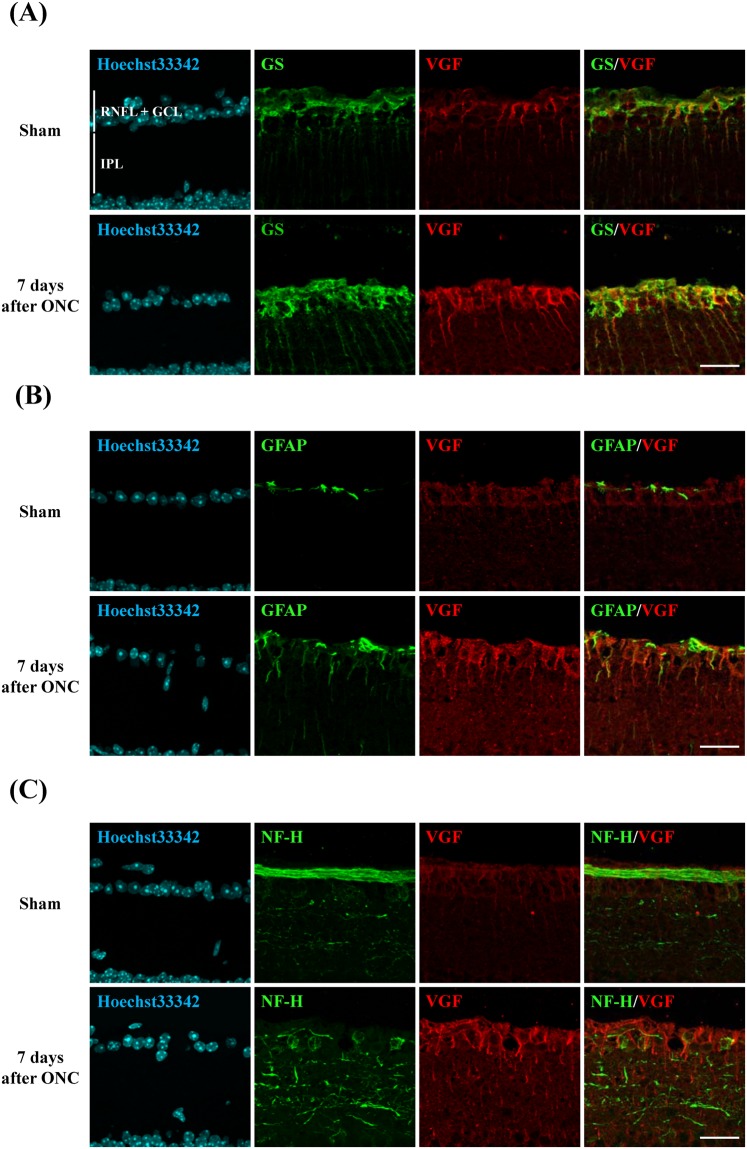
Retinal location of VGF in the sham group and 7 days after ONC group. (**A**,**B**) VGF expression (red) was co-localized with GS^+^ Müller glia (green) and partially co-localized with GFAP^+^ astrocytes (green) in both group. (**C**) VGF expression (red) was localized around the NF-H^+^ nerve fiber (green). Scale bar = 25 μm.

Next, we investigated the site of VGF expression in the different retinal layers by immunostaining with markers for nerve fibers, Müller glia, and astrocytes. This investigation was performed at 7 days after the ONC because the level of VGF is increased in this time. At 7 days, the immunoreactivity was increased throughout the RNFL + GCL and INL and especially around the cells in the GCL. VGF was co-localized with GS, a marker of Müller glia, in both the ONC and control groups (Fig. 2A). VGF was partly co-localized with GFAP, a marker of astrocytes (Fig. 2B). On the other hand, VGF was not co-localized with NF-H, a marker of neuronal axons (Fig. 2C).

### Expression and localization of VGF in optic nerve

**Figure 3:**
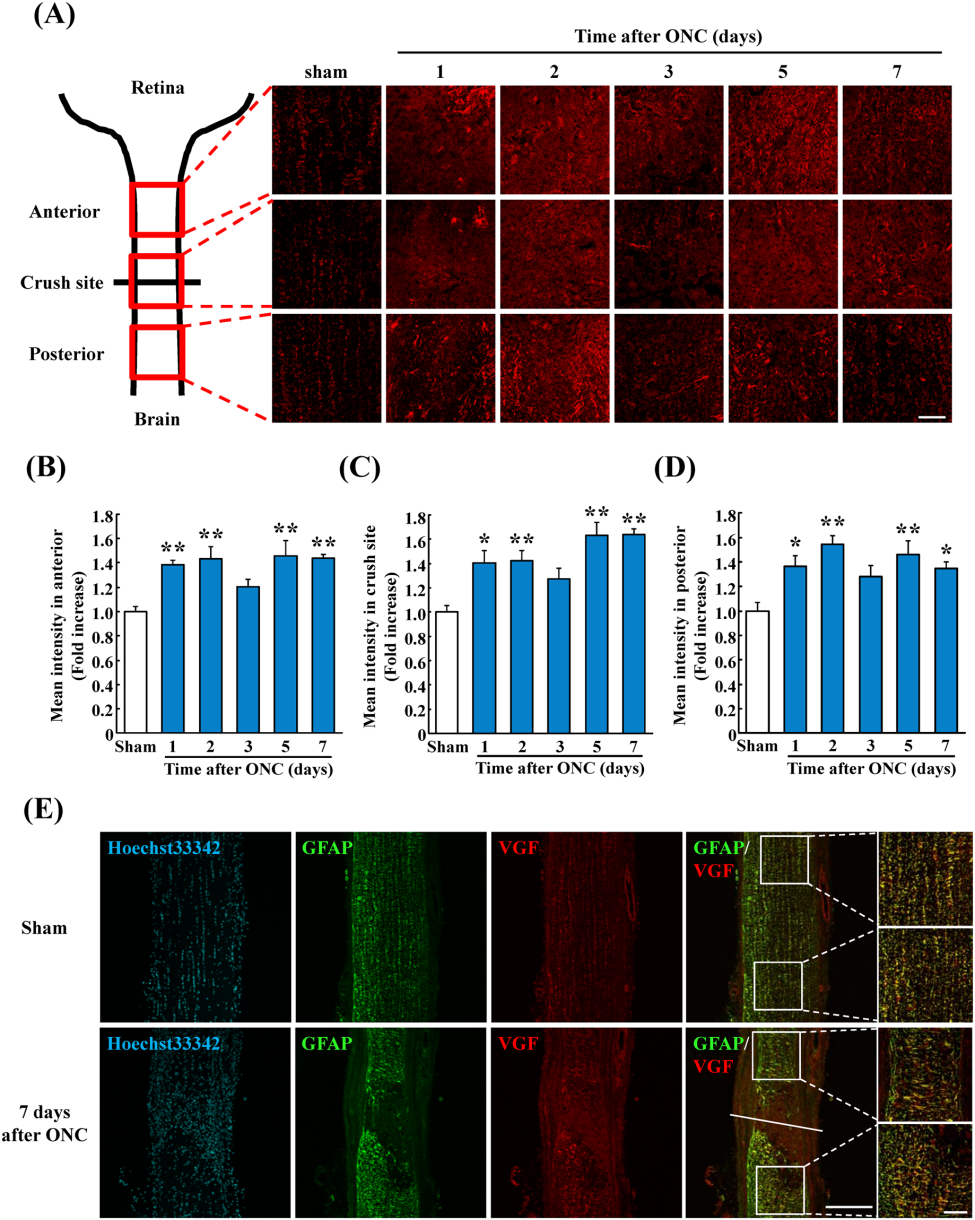
VGF expression and localization in optic nerve. (**A**) Representative images showing the sites of the VGF expression in the optic nerve. The intensity of VGF expression was measured anterior to the crush site (**B**), the crush site (**C**), and posterior to the crush site (**D**). The expression of VGF is significantly higher on 1, 2, 5, and 7 days after the ONC at all 3 areas. (**E**) VGF expression (red) was co-localization with GFAP^+^ astrocytes (green) in both groups. Data are the means ± SEMs (n = 5). *P < 0.05, **P < 0.01 versus sham group (Dunnett’s test). Scale bar = 50 μm.

We determined the level and site of expression of VGF in the crushed optic nerve at the anterior site, the crushed site, and the posterior site at 1, 2, 3, 5, and 7 days after the ONC (Fig. 3A–D). VGF was co-localized with GFAP in both the sham group and the ONC group 7 days at the anterior site, and the posterior site after the ONC (Fig. 3E).

### AQEE-30, a VGF peptide, suppressed loss of RGCs induced by ONC

**Figure 4:**
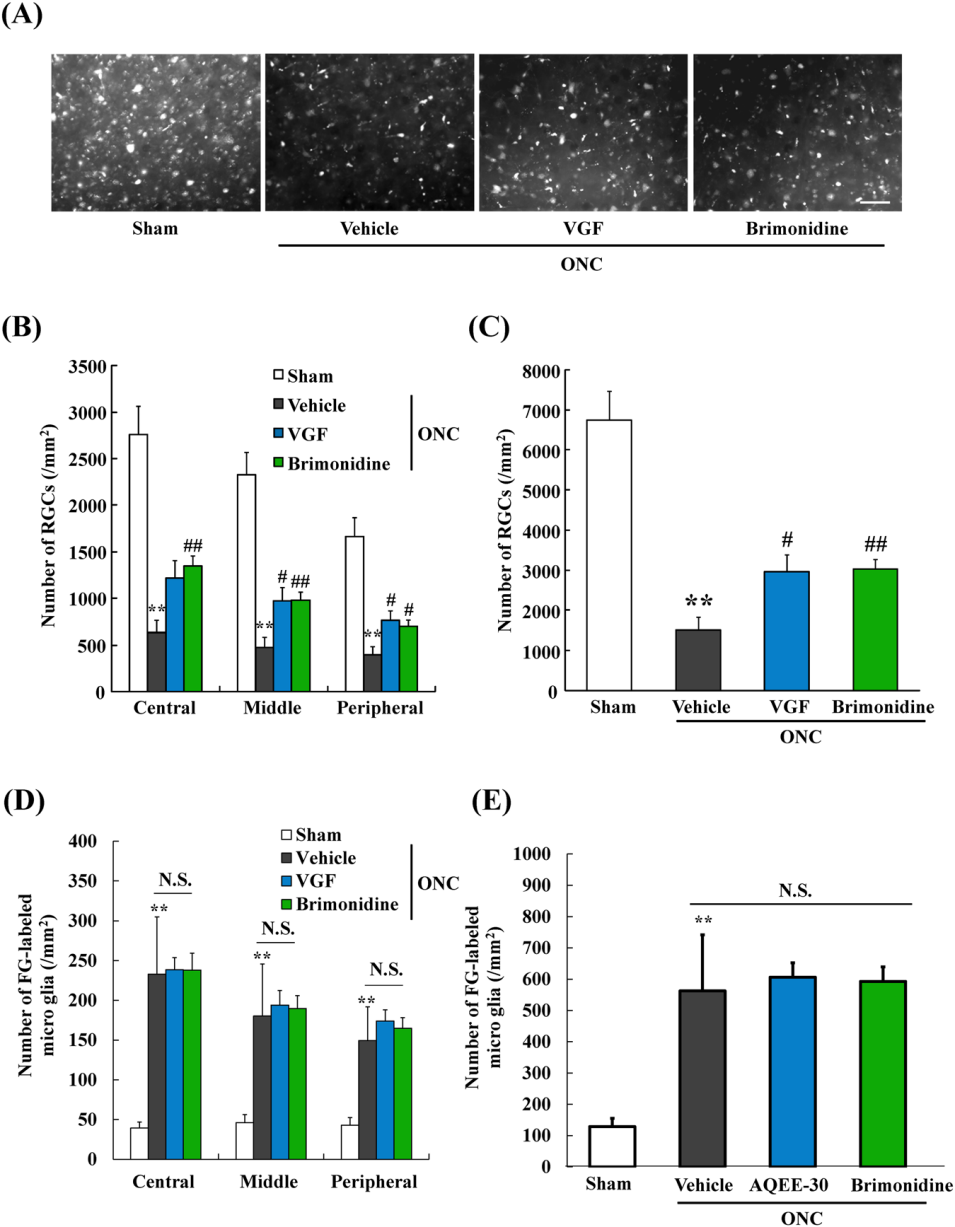
AQEE-30, a VGF peptide, suppresses the loss of retinal ganglion cells (RGCs), but do not change the number of microglia induced by ONC. (**A**) Representative images showing fluorogold-labeled RGCs in flat-mounted retinas at 10 days after the ONC. (**B**) The number of surviving RGCs was counted at the central, middle, and peripheral areas. (**C**) The total number of surviving RGCs is shown. AQEE-30 inhibited the loss of RGCs. Data are the means ± SEMs. (n = 4–12). **P < 0.01 versus sham group (Student’s *t*-test). ^#^*P* < 0.05, ^##^*P* < 0.01 versus vehicle-treated group (Student’s *t*-test). Scale bar = 50 μm. (**D**) The number of FG-labeled microglia was counted at the central, middle, and peripheral areas. (**E**) The total number of FG-labeled microglia is shown. AQEE-30 and Brimonidine did not change the total number of FG-labeled microglia. **P < 0.01 versus sham group (Student’s *t*-test).

We evaluated the effects of AQEE-30 on the loss of RGCs. Representative photomicrographs of fluorescence-labeled RGCs in flat-mounted retinas are shown in Fig. 4A. First, we found that the number of RGCs was significantly decreased in the vehicle-treated ONC group compared with the sham group (Fig. 4B,C). In addition, we used brimonidine which slightly suppresses the reduction of RGCs in the ONC model for the positive control^30^. As the result, we confirmed that brimonidine reduced loss of RGCs in this model. As the previous reports, administration of BDNF promote RGCs survival about 1.5 ~ 2.0 times in the optic nerve injury model^31,32^. Actually, our study showed that brimonidine prevented ONC-induced RGCs loss in the same way. In addition, brimonidine stimulates trophic factor productions including BDNF^33^. Thus, we considered that brimonidine is suitable as a positive control in this ONC model. Using this model, we found that AQEE-30 slightly suppressed the loss of RGCs after the ONC compared to the loss in the vehicle-treated group (Fig. 4B,C). In addition, AQEE-30 and brimonidine treatment did not change the number of FG-labeled microglia (Fig. 4D,E).

### Effects of VGF overexpression on RGCs after ONC

**Figure 5:**
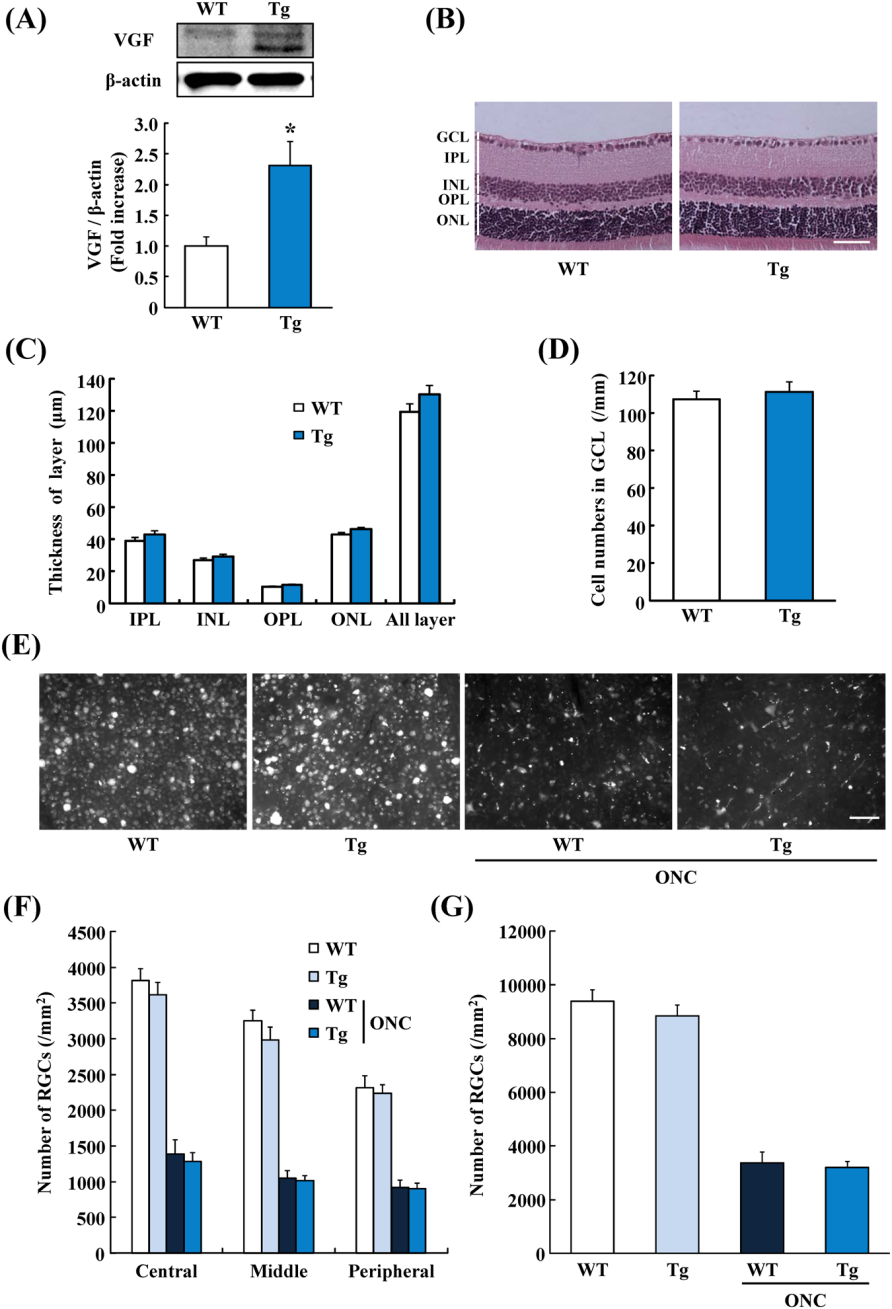
VGF Overexpression of VGF does not inhibit the death of RGCs induced by ONC. (**A**) The expression levels of VGF in the VGF-overexpressing mice (Tg) retina was higher than wildtype mice (WT) retina. Data are the means ± SEMs. (n = 3). *P < 0.05 versus WT group (Student’s *t*-test). (**B**) Representative images of normal retinal structure in WT mice and VGF-overexpressing mice. (**C**) Quantitative data of retinal thickness in normal retina. In each layer, the thickness was not significantly different between WT mice and VGF-overexpressing mice. (**D**) The number of cells in GCL is not significantly different between WT mice and VGF-overexpressing mice. Data are the means ± SEMs. (n = 9–11). Scale bar = 50 μm. (**E**) Representative fluorescence images showing RGCs in the flat-mounted retina at 10 days after the ONC. (**F**) Quantitative data showing the number of fluorogold-labeled RGCs at the central, middle, and peripheral areas. (**G**) Total number of surviving RGCs was measured. Overexpression of VGF did not affect the death of RGCs. Data are the means ± SEMs. (n = 5–10). Scale bar = 50 μm. GCL; ganglion cell layer, IPL; inner plexiform layer, INL; inner nuclear layer, OPL; outer plexiform layer, ONL; outer nuclear layer. The cropped blots are used in this figure. The full-length blots are presented in Supplementary Fig. 2.

We investigated whether the ONC-induced RGCs death would be suppressed in VGF-overexpressing mice. In preliminary studies, we confirmed that the expression of VGF protein in the retina of VGF-overexpressing mice was 2.3 times higher than that of WT mice (Fig. 5A). The retinal structure of VGF-overexpressing mice was not different from that of WT mice (Fig. 5B–D). Ten days after the ONC, the number of RGCs in the VGF-overexpressing mice was not significantly different from that in WT mice (Fig. 5E,G).

### Effects of VGF peptide on RGCs in culture

**Figure 6:**
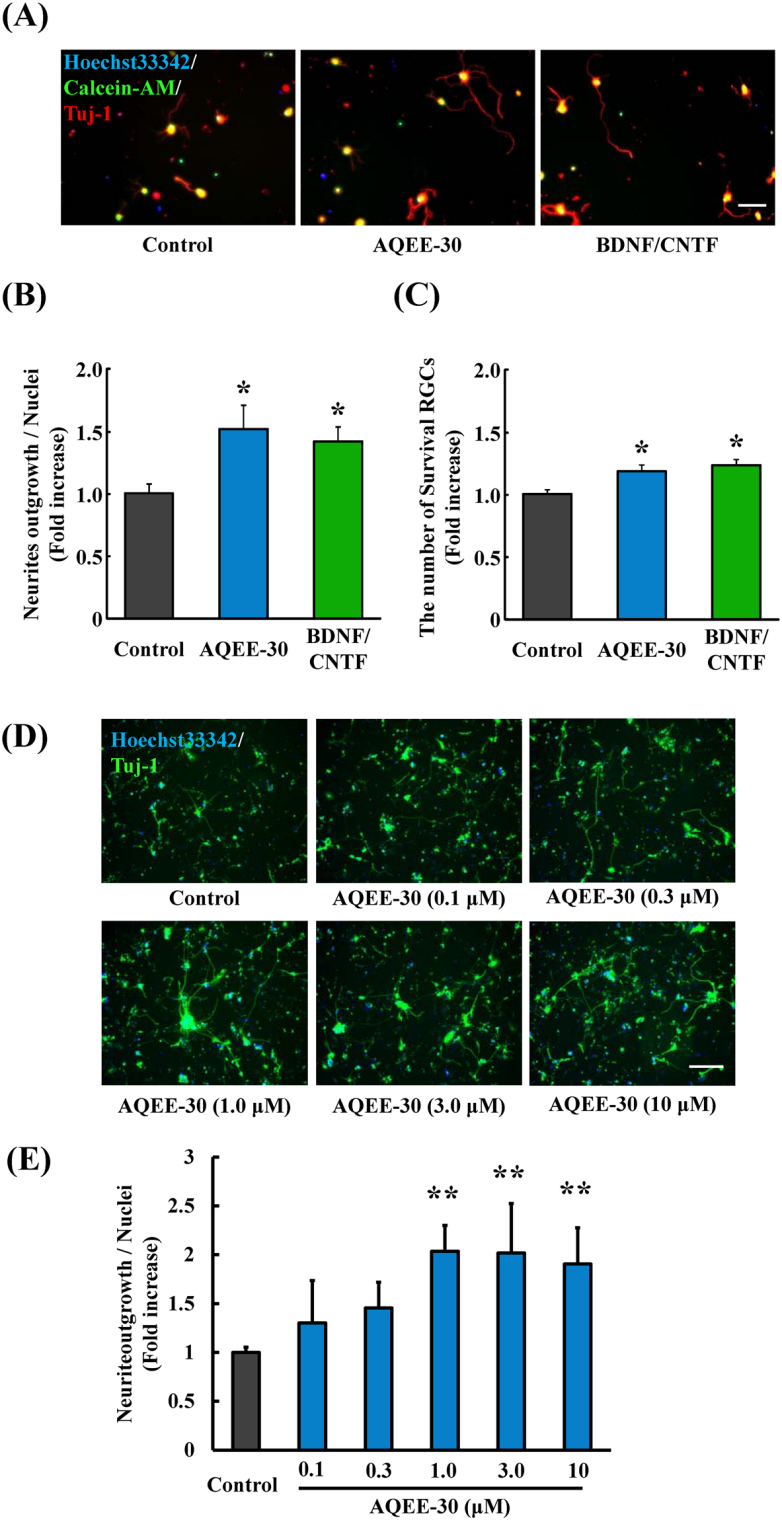
AQEE-30 promotes neurites outgrowth from RGCs *in vitro*. (**A**) Typical images of cultured rat-derived RGCs at 3 days after purification. The neurites are stained green by Calcein-AM^+^ (green) and cells by Tuj-1^+^ (red). (**B**) Quantitative data showing the neurites length of RGCs. AQEE-30 at 1 µM promotes neurites outgrowth in rats-derived RGCs. Data are the means ± SEMs (n = 6). *P < 0.05 versus control group (Student’s *t*-test). Scale bar = 50 μm. (**C**) Quantitative data showing the Survival RGCs. (**D**) Immunostained images of human induced pluripotent stem cells (iPSCs) derived RGCs culture at 50 days after the neuronal induction. The neurites of the cells are stained green by Tuj-1^+^. (**E**) AQEE-30 at 1 µM to 10 µM also promotes neurite outgrowth in human iPSCs-derived RGCs in a concentration dependent manner. Data are the means ± SEMs. (n = 4). **P < 0.01 versus control group (Dunnett’s test).

Finally, we assessed the direct effects of AQEE-30 against RGCs by using rat-derived RGCs and human iPSCs-derived RGCs in culture. In this investigation, BDNF and CNTF were used as positive control because these factors are known to promote outgrowth of RGCs neurites. Similarly, the neurites outgrowth and the number of survival rat-derived RGCs was promoted by AQEE-30 treatment compared with the control group (Fig. 6A–C). For the human iPSCs-derived RGCs, we evaluated whether AQEE-30 also promoted neurites outgrowth in a concentration dependent manner. As the result, AQEE-30 enhanced neurite outgrowth in a concentration dependent manner and suggesting that 1 µM AQEE-30 is the minimal effective concentration (Fig. 6D,E). Thus, it is estimated that 1 µM AQEE-30 would be needed to expect the neuroprotective effect in the injected retinas. In addition, high-concentration of AQEE-30 treatment (3 µM and 10 µM) did not show toxicity, suggesting that range of AQEE-30 concentration within 1–10 µM would not be toxic for RGCs.

## Discussion

The results showed that VGF was associated with the retinal and optic nerve degeneration induced by the ONC, and that AQEE-30 had neuroprotective effects against the ONC. We found that the expression of the mRNA of the *Vgf* gene and the VGF protein were upregulated after the ONC. In addition, immunohistochemistry showed that VGF was expressed in the glial cells but not in neuronal axons. Moreover, AQEE-30 slightly suppressed the reduction of the number of RGCs after the ONC, and it also promoted neurite outgrowth in rat-derived RGCs and human iPSCs-derived RGCs.

Neurotrophic factors are known to be secreted peptides that influence neuronal survival, development, and function. Although previous studies have shown that neurotrophic factors such as BDNF, NGF, CNTF, glial cell-line derived neurotrophic factor (GDNF), and basic fibroblast growth factor (bFGF) were found to promote RGCs survival^34–38^, the application of these factors as medications has not been realized. The difficulties with these factors are the low bioavailability and the necessity of frequent administration to have an effect due to their large molecular weight and short half-life^37^. Similar to BDNF, the effects of VGF peptides including those promoting neuronal survival and regulating synaptic plasticity are associated with BNDF-TrkB mediated signaling pathway in brain neuronal cells^38,39^. Conversely, the molecular weight of VGF peptides is much lower than that of BDNF. Therefore, VGF should be considered as a potential agent to treat axonal injury. However, the molecular function of VGF has not been determined in retina although the function of VGF in brain is known. In this study, the mRNA of *Vgf* was increased in the retina at 2, 3, and 5 days after the ONC. Furthermore, the expression of the VGF protein was upregulated 7 days after the ONC especially in the RNFL + GCL. The results of previous studies have indicated that the mRNA levels of both BDNF and bFGF were increased at 3 days after optic nerve transaction^40,41^. Moreover, a reduction in the number of RGCs, which is inhibited by neurotrophic factors, occurred rapidly from 3 to 7 days after the ONC^42^. Therefore, these findings suggest that the mRNA of *Vgf* and VGF protein increased with the loss of RGCs induced by the ONC as well as the neurotrophic factors.

VGF is expressed in neuronal cells such as the hippocampal neurons and dorsal root ganglion neurons in the central nerve systems (CNS)^43–45^. According to these reports, VGF also seemed to be localized in the retinal neurons including the RGCs before investigation. Interestingly, VGF was localized in glial cells including the Müller glia and astrocytes but not in neuronal axons. Glial cells such as astrocytes and Müller glia are known to provide trophic support for retinal neurons^46,47^. For example, Müller glia produce neurotrophic factors including BDNF^48^. Importantly, glial cells undergo morphological changes and alterations of their gene expression after injury or disease which is called reactive gliosis^49,50^. Genomic analysis showed that the gene of *Vgf* and *Bdnf* were upregulated after reactive gliosis in the mouse brain^51^. Additionally, microglia were reported to be the source of BDNF for neurons and contribute to neuroprotection and functional recovery^52,53^. In the ONC model, reactive gliosis occurred in the retina^54^, and an activation of astrocytes and migration of microglia were observed in the optic nerve after the ONC^55^. We also observed changes of the astrocyte-like immunoreactivity in the retina and the optic nerve after the ONC along with an increase in the VGF expression after the ONC. Thus, these findings suggest that an increase of VGF occurred with the reactive gliosis. In Fig. 3A–D, VGF increased except for 3 days in the optic nerve after the ONC. Previous reports indicated that astrocytes, which expressed VGF protein, started to disappear from the crush site as early as 2 days after the ONC^56^. On the other hand, activation of astrocytes and scar forming was occurred at 7 days after the ONC in the optic nerve^13^. Therefore, we estimated that VGF might not increase at 3 days after the ONC and might increase from 5 days to 7 days after the ONC.

Although the level of endogenous VGF was increased, the number of RGCs was reduced. The reason for this is that the time when the expression of VGF was upregulated after the ONC was considered to be too late for protecting RGCs. Because this time coincides with the late stage of reducing the number of RGCs induced by the ONC, a supply of VGF is thought to be required at the early stage. In fact, although it was reported that CNTF and bFGF was upregulated in retina 7 days after the ONC^14,57^, the administration of these neurotrophic factors attenuated the loss of RGCs^36^.

To administrate VGF, we considered what VGF peptide is appropriate. The processed VGF peptides can be divided into two groups; one is the peptides including TLQP-21 and AQEE-30 that are generated mainly by cleavage of VGF C-terminal, and the other including NERP-1 and NERP-2. It has been reported that NERP-1 and NERP-2 regulate water homeostasis and secretion of vasopressin in the hypothalamus^25,58^. On the other hands, TLQP-21 and AQEE-30 are involved in neuronal activity^59,60^. It was recently shown that AQEE-30 but not TLQP-21 has neuroprotective effects against the Huntington’s disease model^27^. Huntington’s disease is a neurodegenerative disease that results in the death of neuronal cells within the basal ganglia and the cerebral cortex. In primary retinal cells, the neuroprotective effect of VGF against RGCs has been shown^29^. Therefore, AQEE-30 is most likely involved in the neuroprotective effects on the RGCs in the ONC model.

As this prediction, VGF treatment by an intravitreal injection of AQEE-30, inhibited the reduction of the RGCs. Additionally, AQEE-30 treatment promoted the outgrowth of neurites in RGCs cultures. According to these findings, it can be inferred that AQEE-30 has direct effects on RGCs. Consequently, AQEE-30 has promise for protecting RGCs, although the neuroprotective mechanism of AQEE-30 has not been revealed. To explore the potency of AQEE-30 for a neuroprotective agent, more studies are needed. For example, AQEE-30 treatment did not change the number of FG-labeled microglia in this study. Generally, the number of FG-labeled microglia, which approximately correlate with phagocytic activity of microglial cells and FG-labeled RGCs are inverse correlation at 10 days from ONC^61^. Considering this study, FG-labeled microglia are expected to decrease in the VGF and brimonidine treatment group because VGF and brimonidine inhibited loss of RGCs. However, FG-labeled microglia did not change in these groups compared with the vehicle group. We did not determine why FG-labeled microglia did not change by administration of VGF and brimonidine in this study. As one of several possibilities, we possibly speculate the involvement of BDNF on microglia. The reason why is that VGF and brimonidine have the potential to activate BDNF-TrkB receptor-mediated signaling pathways^33,38,39,62^. Moreover, BDNF enhances microglial phagocytic activity^63,64^. Thus, we cannot exclude the possibility that VGF and brimonidine would enhance microglial phagocytic activity via increasing BDNF production.

In spite of the neuroprotective activity of AQEE-30 for RGCs, VGF overexpression did not protect RGCs death induced by the ONC. In the VGF-overexpressing mice, the retinal VGF expression was about 2.4 fold higher than that of WT mice. Regarding the amount of endogenous VGF, previous reports have indicated that the concentration of VGF peptides in mouse plasma was 5 pmol/ml^65^. According to this report, it is assumed that VGF peptides expression in the VGF-overexpressing mouse plasma was about 11 pmol/ml. In contrast, the treating concentration of AQEE-30 was 50 µmol/L. For this reason, the endogenous VGF in VGF-overexpressing mice retina was quiet low. As a result, the retinal expression levels of VGF in VGF-overexpressing mice might not be enough to protect the RGCs.

In conclusion, we identified the close association between VGF and neuronal degeneration induced by the ONC and neuroprotective effects of VGF peptides. Therefore, treatments targeting the VGF should be considered in RGCs degeneration.

## Methods

### Ethics Statement

The procedures used in the experiments on human induced pluripotent stem cells (iPSCs; 201B7 line) adhered to the tenets of the Declaration of Helsinki, and were approved by the Ethics Review Committee of the National Hospital Organization, Nagara Medical Center and Gifu Pharmaceutical University. The 201B7 line was generated from adult human dermal fibroblasts which was purchased from Cell Applications, Inc (San Diego, CA, USA)^66^. This cell line was kindly provided, and we used this cell line based on the contract with the Center for iPS Cell Research and Application, Kyoto University^66^.

All of the procedures used in the animal experiments conformed to the guidelines of the Association for Research in Vision and Ophthalmology (ARVO) Statement for the Use of Animals in Ophthalmic and Vision Research. The Institutional Animal Care and Use Committee and the Institutional Biosafety Committee of Gifu Pharmaceutical University approved and monitored all animal experiments.

### Animals

Eight-week-old male ddY albino mice (35–40 g body weight) and adult Sprague-Dawley rats (250–350 g body weight) were purchased from Japan SLC (Hamamatsu, Japan). Neonatal rats were obtained from breeding the purchased rats. Fifteen-weeks-old male VGF-overexpressing mice (25–40 g body weight) were generated as described in detail^67^. Animals were housed under 12 h:12 h light:dark lighting conditions with a standard diet (CLEA Japan, Inc., Tokyo, Japan).

### Optic nerve crush model

The ONC was performed as described in detail^68,69^. Briefly, mice were anesthetized with a mixture of 120 mg/kg ketamine (Daiichi-Sankyo, Tokyo, Japan) and 6 mg/kg xylazine (Bayer Health Care, Tokyo, Japan) for the surgery. The optic nerve of the left eye was exposed and crushed at 0.5–1.5 mm posterior to the eye. In sham group, only the exposure of optic nerve was performed. After the ONC, ofloxacin ointment (Daiichi-Sankyo) was applied topically.

### Labeling and histological analysis of retinal ganglion cells (RGC)

To determine the number of RGCs surviving after the ONC, the RGCs were retrogradely labeled with fluorogold three days before the ONC as described in detail^68^. Briefly, 1 µL of 4% fluorogold (Biotium, Hayward, CA, USA) was injected at a depth of 2 mm from the brain surface into the superior colliculus under anesthesia.

Ten days after the ONC, mice were euthanized by cervical dislocation, and the eyes were enucleated. The eyes were fixed in 4% paraformaldehyde (PFA) at 4 °C, and after 6 to 7 h, the retinas were isolated from the retinal pigment epithelium, and flat-mounted on glass slides. To quantify the number of RGCs, the retinas were examined with a BX50 fluorescence microscope and photographed with a DP30BP camera (Olympus, Tokyo, Japan). Fluorescent images were produced using the MetaMorph software (Universal Imaging Corp., Downingtown, PA, USA). To determine the percentage of RGCs surviving, the number of stained RGCs was counted manually at 0.5 mm (central), 1.5 mm (middle) and at 2.5 mm (peripheral) from the optic nerve head. We distinguished FG-labeled RGCs from FG-labeled microglia as the previous reports^70–73^. Briefly, FG-labeled elongated or irregularly shaped cells are regarded as microglia. These cells are both larger and smaller than RGCs. The averages of 12 counts/retina were used for the statistical analyses.

### Drug applications after optic nerve crush (ONC)

AQEE-30 (Medical & Biological Laboratories Co., Ltd, Aichi, Japan), a VGF peptide, was dissolved in phosphate buffered saline (PBS) to a concentration of 50 µM/2 µl. PBS was injected into the control eyes. Brimonidine tartrate (Sigma-Aldrich, St. Louis, MO, USA) was dissolved in saline and used as a positive control. AQEE-30 (50 µM/2 µl/eye) was administered by an intravitreal injection immediately, 2, and 5 days after the ONC. Brimonidine tartrate (100 µg/kg) was administrated by an intraperitoneal injection immediately after the ONC.

### RNA extraction and real-time PCR

Mice were killed on 1, 2, 3, 5, and 7 days after the ONC, and the eyes were quickly removed. The retinas were carefully isolated and frozen in liquid nitrogen. The isolation of the RNA from the retinas and quantification of gene expression were performed as reported in detail^63^. The expression of glyceraldehyde-3-phosphate dehydrogenase (*Gapdh*) was used for the internal control. The sequences of the primers were;

for *Vgf*,

5′-CAGGCTCGAATGTCCGAAAG-3′ (forward) and

5′-CTTGGATAAGGGTGTCAAAGTCTCA-3′ (reverse)

for *Gapdh*,

5′-TCTGCAAGAGACTTCCATCCAGT-3′ (forward) and

5′-TCTGCAACTGCATCATCGTTGT-3′ (reverse).

### Immunostaining

Sections were prepared and stained as reported in detail^74^. The following primary antibodies were used: rabbit anti-VGF polyclonal antibody (1:50; Abcam; Cambridge, MA, USA), mouse anti-glial fibrillary acidic protein (GFAP) monoclonal antibody (1:500; Millipore; Bedford, MA, USA), mouse anti-glutamine synthetase (GS) monoclonal antibody (1:1000; Millipore), and mouse anti-neurofilament-Heavy (NF-H) monoclonal antibody (1:1000; Millipore). Alexa Fluor®488 goat anti-rabbit IgG, Alexa Fluor®488 goat anti-mouse IgG, and Alexa Fluor®546 donkey anti-rabbit IgG (1:1000; Invitrogen) were used as the secondary antibodies. The stained sections were examined with a confocal microscope (FLUOVIEW FV10i; Olympus), and photographs were taken at 500 μm superior to the optic nerve head.

Photographs of the optic nerve were taken at 3 areas; 250 μm anterior to the crush site (anterior), 250 μm posterior to the crush site (posterior), and at the crush site. The immunostained sections were used to determine the site of expression of VGF in the retina and optic nerve. The intensity of the VGF was measured with ImageJ software (National Institutes of Health, Bethesda, MD). The mean of 3 sections were used for the statistical analyses. Different retinal layers were investigated; the retina from the inner edge of the outer nuclear layer to the internal limiting membrane, the retinal nerve fiber layer plus ganglion cell layer (RNFL + GCL), and the inner plexiform layer (IPL) were analyzed.

### Western blot analysis

Retinal samples were prepared as described in detail^68^, and western blot analysis was performed according to a reported protocol^67^. Goat anti-VGF polyclonal antibody (1:400 dilution; Santa Cruz Biotechnologies, CA, USA) and mouse anti-β-actin monoclonal antibody (1:1000; Sigma-Aldrich) were used as the primary antibodies for immunoblotting. Goat anti-mouse horseradish peroxidase-conjugated IgG (1:1000) was used as a secondary antibody. Immunoreactive bands were made visible by Immuno Star^®^ LD (Wako), and measured with the LAS-4000 Mini kit (Fuji Film Co., Ltd., Tokyo, Tokyo).

### Histological analysis

Eyes were isolated from VGF-overexpressing and wildtype (WT) mice, and immersed in 4% PFA for 24 h at 4 °C. The fixed eyes were dehydrated in ethanol, cleared with xylene, and embedded in paraffin. Six 5 μm sections were cut through the optic nerve head, and the sections were stained with hematoxylin and eosin. Light microscope images were photographed (Micro Publisher 5.0 RTV; QIMAGING, Surrey, BC, Canada). The cells in the RGC layer and the thicknesses of the IPL, inner nuclear layer (INL), outer plexiform layer (OPL), and outer nuclear layer (ONL) were measured between 375 and 625 μm from the optic disc. The results from 3 sections which were selected randomly from the six sections were averaged for each eye.

### Purified rats RGCs cultures and drug treatment

Purified rat RGCs were isolated on postnatal day 5 from retinas that were dissociated with MACS dissociation kit (Miltenyi Biotec, Bergisch Gladbach, Germany). The RGCs were isolated with the MACS RGC isolation kit (Miltenyi Biotec). The dissociation of the retinas and isolating the RGCs were performed following the manufacturer’s instructions. Cells were plated at about 2,500 cells/well in 96-well plates and cultured in serum-free neurobasal medium (Invitrogen) supplemented with 2% B-27 supplement (Invitrogen), 1 mM pyruvate acids (Sigma-Aldrich), 60 ng/ml *N*-acetylcysteine (Wako), 10 µM forskolin (Wako), 2mM L-glutamine (Nacalai Tesque, Kyoto, Japan), 40 ng/ml triiodothyronin (Sigma-Aldrich), 5 µg/ml insulin (Sigma-Aldrich), 100 U/ml penicillin (Meiji Seika Pharma, Tokyo, Japan), and 50 mg/ml streptomycin (Meiji Seika Pharma). The plates had been coated with 0.05 mg/ml poly-D-lysine (Sigma-Aldrich) overnight, rinsed three times with PBS, and then coated for 2 hours with 1 µg/ml of laminin (Corning, Corning, NY, USA).

After incubating for 24 h, the RGCs were exposed to 3 µM AQEE-30 or a combination of 50 ng/ml recombinant human BDNF (Miltenyi Biotec) and 10 ng/ml recombinant human CNTF (Miltenyi Biotec) for 48 h. Then, 2 mM calcein-AM (Dojindo Laboratories, Kumamoto, Japan) was added to label the surviving RGCs.

### Induction of RGCs from human induced pluripotent stem cells (iPSCs) and drug treatments

The culture of isolated iPSCs and production of embryoid bodies with the quick reaggregation (SFEBq) method was performed as described in detail^75,76^. To create mimics of normal RGCs, we use a reported modified protocol^77–79^. On the day after beginning the culture, the culture medium was changed to the differentiation medium consisting of Dulbecco’s Modified Eagle Medium/F12 (Invitrogen), 1% N2 supplement (Invitrogen), B27 supplement, L-glutamine, 500 U/ml penicillin/streptomycin (Invitrogen), 2 μM dorsomorphin (Sigma-Aldrich), 10 ng/ml human Dickkopf1 (R&D systems, Minneapolis, USA), 10 ng/ml insulin-like growth factor-1 (R&D systems), and 10 ng/ml bFGF (R&D systems) for 7 days. The neuronal precursor cells were cultured in a differentiation medium containing 10 μM of N-[(3,5-Difluorophenyl)acetyl)]-L-alanyl-2-phenylglycine-1,1-dimethyl ester (DAPT; Tocris Bioscience, Avonmouth, UK) for a further 7 days. At the final stage, all cells except the embryoid bodies were reseeded at 5,000 cells/well into Matrigel-coated 96-well plates and cultured with the addition of 2 ng/ml acidic fibroblast growth factor (R&D Systems) to the differentiation medium for 11 days. The medium was changed every 2 or 3 days for all differentiated stages. Differentiated RGCs were exposed to 1 μM AQEE-30 or 100 ng/ml BDNF (R&D systems) for 16 days with the medium changed every 3 days.

### Immunocytochemistry and neurite outgrowth evaluation

The plated cells were fixed in 4% PFA for 15 min at room temperature. The cells were blocked with non-immune serum for 30 min at room temperature and then incubated with rabbit anti-class III β-tubulin (Tuj-1) monoclonal antibody (1:1000; Biolegend, San Diego, CA, USA) overnight at 4 °C. On the following day, the cells were labeled with Alexa Fluor®546 donkey anti-rabbit IgG (1:1000) for 1 h. The nuclei were stained with Hoechst 33342, and the sections were mounted in Fluoromount.

To evaluate the rat-derived RGCs, 9 photographs/well were taken at random sites with a fluorescence microscope (BZ-X710; Keyence, Kyoto, Japan). For this assay, calcein-AM and Tuj-1 positive-RGC were defined as live RGCs, and the neurite length of these cells were measured semi-automatically with the BZ-X analyzer (Keyence).

For the analysis of human iPSCs-derived RGC, 5 images/well were taken with a fluorescence microscope (BZ-9000; Keyence), and the lengths of the neurites of the Tuj-1 positive cells were measured semi-automatically.

### Statistical analysis

Data are presented as the means ± standard error of the means (SEMs). Statistical comparisons were made by Student’s *t*-tests or Dunnett’s tests with the SPSS Statistics software (IBM, Armonk, NY, USA). A *P* value < 0.05 was taken to be statistically significant.

## Electronic supplementary material

Supplemental figure
