# Supplementary material for: VGF nerve growth factor inducible is involved in retinal ganglion cells death induced by optic nerve crush
Source: Sci Rep. 2018 Nov 6;8:16443. doi: 10.1038/s41598-018-34585-3 (PMC6219571; doi:10.1038/s41598-018-34585-3)
Supplement: Supplementary file 1 — Supplemental figure [file 41598_2018_34585_MOESM1_ESM.pdf]

Supplementary information

VGF nerve growth factor inducible is involved in retinal ganglion cells death induced by optic nerve crush

Hiroto Takeuchi[1#], Satoshi Inagaki[1, 2#], Wataru Morozumi[1], Yukimichi Nakano[1], Yuki Inoue[1], Yoshiki Kuse[1], Takahiro Mizoguchi[1], Shinsuke Nakamura[1], Michinori Funato[2], Hideo Kaneko[2], Hideaki Hara[1], Masamitsu Shimazawa[1]*

[1]Department of Biofunctional Evaluation, Molecular Pharmacology, Gifu Pharmaceutical University.

[2]Department of Clinical Research, National Hospital Organization, Nagara Medical Center, Gifu, Japan.

[#]Contributed equally

*Corresponding Author: M. Shimazawa, PhD, Molecular Pharmacology, Department of Biofunctional Evaluation, Gifu Pharmaceutical University, 1-25-4 Daigaku-nishi, Gifu 501-1196, Japan.

E-mail: shimazawa@gifu-pu.ac.jp,

TEL/FAX: +81-58-230-8126

Supplementary Figure 1

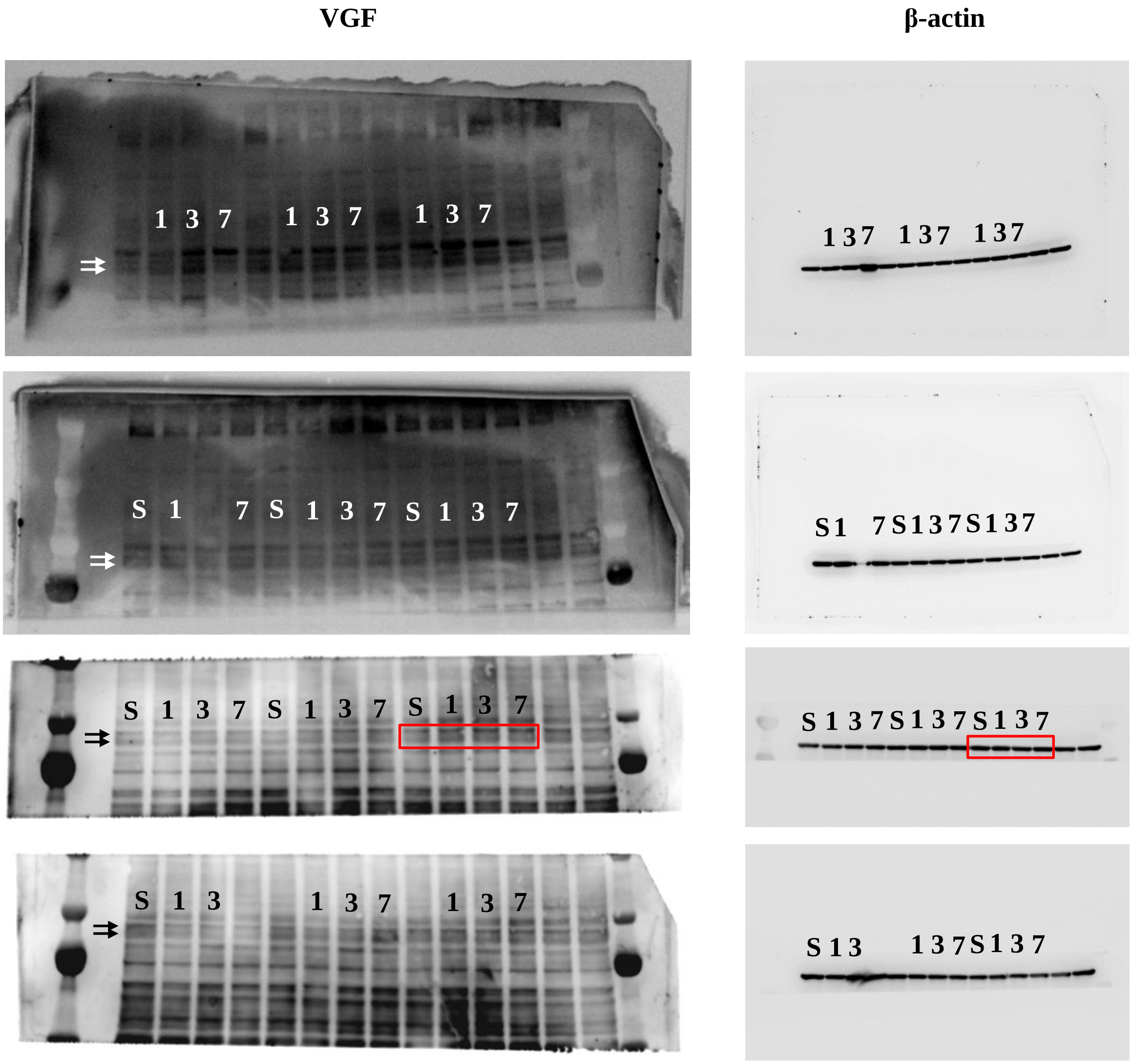

Supplementary Figure 1. The full-length blots of the expression of VGF protein after optic nerve crush in retina using western blotting.

Immunoblotted images to determine the level of expression of VGF protein in whole retina after the optic nerve crush (ONC). Arrows indicate the site of VGF protein expression. The blots in squares are used for the cropped blots in Figure 1. S: Sham; 1: 1 day after ONC; 3: 3 days after ONC; 7: 7 days after ONC.

Supplementary Figure 2

VGF

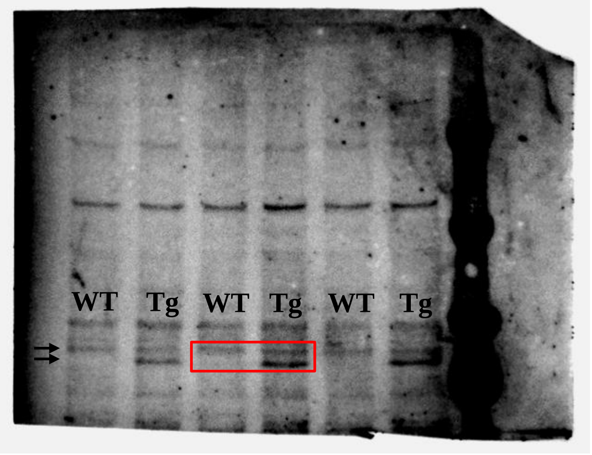

β-actin

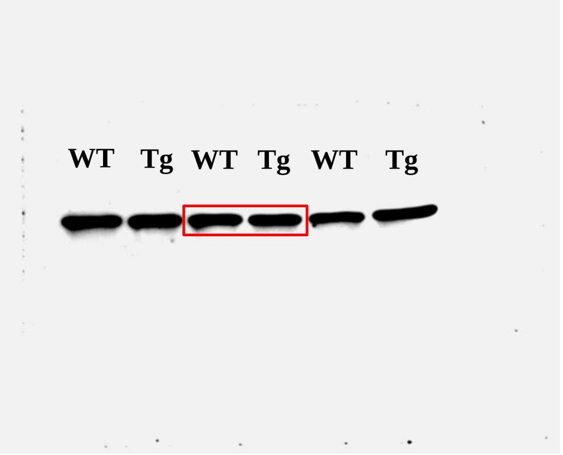

Supplementary Figure 2. The full-length blots of the expression of VGF protein in the VGF-overexpressing mice retina and wildtype mice retina using western blotting.

Immunoblotted images to confirm the level of expression of VGF protein in the VGF-overexpressing mice retina and wildtype mice retina. Arrows indicate the site of VGF protein expression in the membrane. The blots in squares are used for the cropped blots in Figure 5. WT: wildtype; Tg: VGF-overexpressing.
